# Minor trauma triggering cervicofacial necrotizing fasciitis from odontogenic abscess

**DOI:** 10.4103/0974-2700.43197

**Published:** 2008

**Authors:** Shraddha Jain, Prakash S Nagpure, Roohie Singh, Deepika Garg

**Affiliations:** Department of Otorhinolaryngology and HNS, Mahatma Gandhi Institute of Medical Sciences, Sewagram, Maharashtra, India

**Keywords:** Face, fasciitis, necrotizing, tooth diseases/complications, trauma

## Abstract

Necrotizing fasciitis (NF) of the face and neck is a very rare complication of dental infection. Otolaryngologists and dentists should be familiar with this condition because of its similarity to odontogenic deep neck space infection in the initial stages, its rapid spread, and its life-threatening potential. Trauma has been reported to be an important predisposing factor for NF of the face. In this paper, we describe the presentation and treatment of a 62-year-old man who developed NF of the face and neck following bilateral odontogenic deep neck space abscesses. The disease progressed rapidly, with necrosis of the skin, after the patient inflicted minor trauma in the form of application of heated medicinal leaves. The organism isolated in culture from pus was *Acinetobacter sp*. The comorbid conditions in our patient were anemia and chronic alcoholism. The patient was managed by immediate and repeated extensive debridements and split-skin grafting.

## INTRODUCTION

Necrotizing fasciitis (NF) is a rapidly spreading infection involving the superficial fat and fascial layers; it initially spares skin and muscle. Cervicofacial NF is a rare but potentially fatal disease if not diagnosed and treated in the early stages. In the initial stages, before necrosis is seen, the infection spreads in the subcutaneous tissues and may appear as a routine odontogenic deep neck space abscess. Delay in diagnosis leads to increase in the area of necrosis, with a resulting increase in cosmetic deformity and life-threatening complications.

The condition can result from dental (dental abscess, gingivitis, pulpitis, etc.),[1–3] sinus,[4] peritonsillar,[5,6] and salivary gland[7] infections, or from infections secondary to surgery,[8] insect bites, or trauma.[9] Dental infections are the most common etiologic factor, followed by trauma, peritonsillar and pharyngeal abscesses, and osteoradionecrosis. The causative agents have classically been described as being group A beta-hemolytic streptococci, staphylococci, and obligate anaerobic bacteria.[2,10]

In this report we present a rare case of bilateral cervicofacial NF that was caused by *Acinetobacter* bacillus and was secondary to bilateral odontogenic deep neck space abscesses that was triggered by minor trauma in a 62-year-old male.

## CASE REPORT

A 62-year-old male presented to the otolaryngology department of our hospital with a 15-day history of generalized dental pain and bilateral facial swelling, pain, trismus, and fever; these symptoms had begun 1 week after a toothache. The facial swelling first appeared on the left side and then, on the same day, on the right side. The patient had applied heated medicinal leaves on both cheeks, following which he developed a blackish discoloration and swelling of the left cheek. This rapidly progressed to the right side to involve the neck and upper chest and was associated with redness, areas of bluish discoloration, and pain of the skin. Some skin over the face and neck underwent necrosis, with the formation of a yellowish slough and discharge of pus externally and intraorally. These changes occurred very rapidly over 2 days.

The patient was addicted to alcohol and was a chronic smoker. His hemoglobin was 7.5 gm/dl; TLC revealed leucocytosis of 12,300/mm^3^ and DLC showed a predominance of poymorphs (83%). Blood sugar level and liver and renal functions were normal. ELISA for HIV was negative.

**Figure 1:**
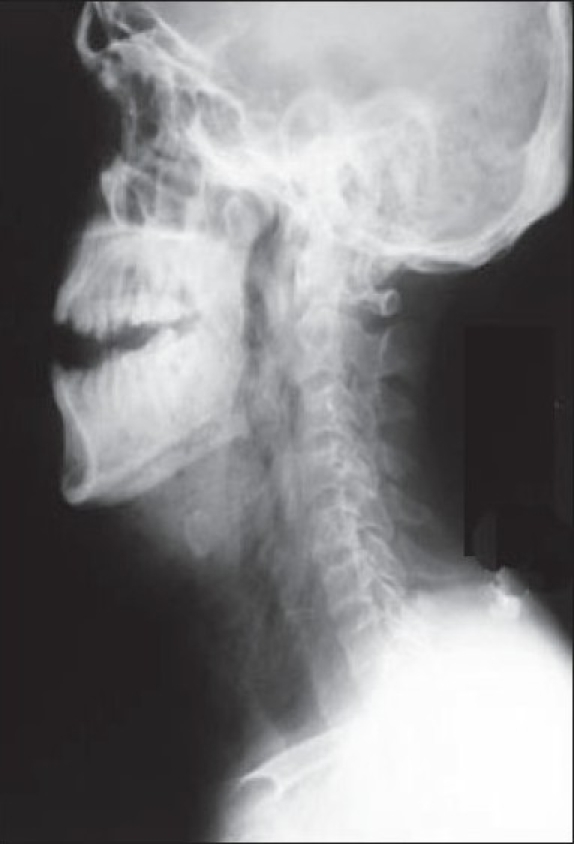
X-ray showing presence of subcutaneous gas formation

Clinical and radiographic examination revealed bilateral parotid and buccal space pus collection with right submandibular and cervical involvement; subcutaneous gas formation was also evident [Figure 1].

**Figure 2:**
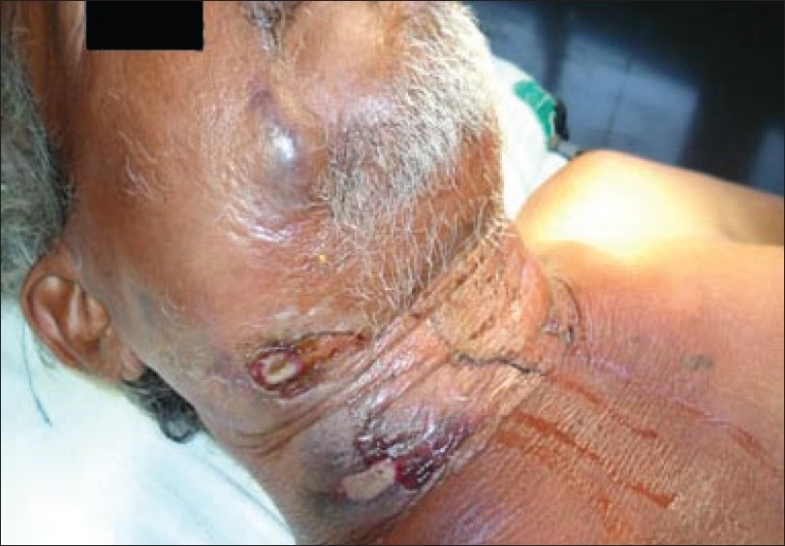
Areas of initial discoloration, oozing, and necrosis in the right cervicofacial region

The parapharyngeal spaces were clear and there was no airway compromise. The necrotic regions were in the submandibular region and above the clavicle on the right side [Figure 2].

**Figure 3:**
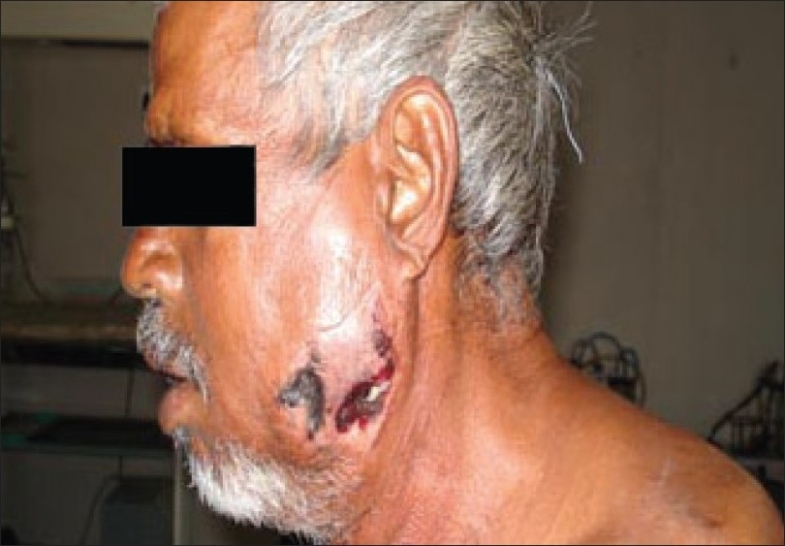
Blackish discoloration over the left cheek

The skin showed changes that ranged from erythema and patchy areas of bluish discoloration to frank necrosis and sloughing with oozing of pus. Marked crepitus was noted extending from the zygomatic region to the neck and upper chest. On the left side, there was a blackish discoloration over the cheek [Figure 3].

**Figure 4:**
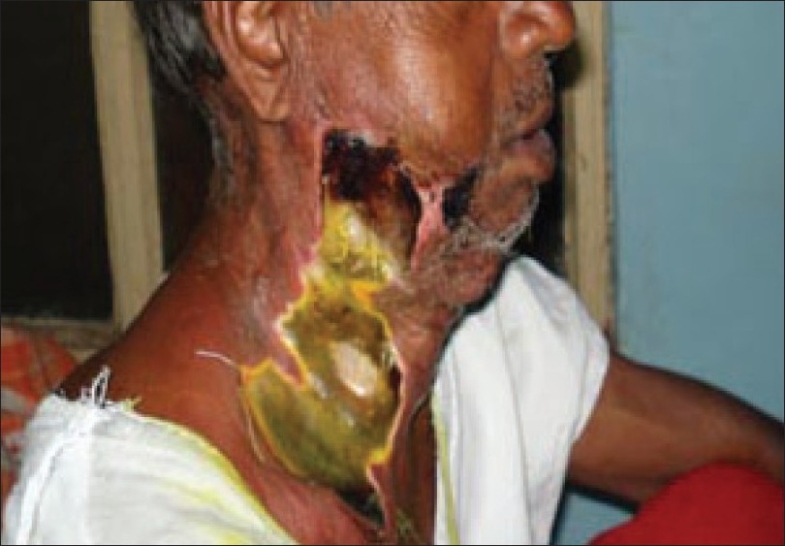
Increasing area of necrosis of cervicofacial skin and subcutaneous tissue on right side

The patient had trismus and oral cavity examination revealed generalized periodontal disease with pulp exposure and pus collection. He was taken to the operating room for immediate incision and drainage of the abscess under general anesthesia with endotracheal intubation. Incision and drainage of bilateral buccal and right cervical abscesses was done, draining approximately 20 cc of foul-smelling pus which, along with multiple tissue samples, was sent for aerobic, anaerobic, and fungal cultures. The culture revealed *Acinetobacter sp.* Immediate complete debridement of all necrotic tissue was performed until bleeding tissue was encountered. The areas of debridement had to be extended on the following days as the zones of involvement and necrosis were seen to increase [Figure 4]. Subsequent culture from different tissue samples sent during the repeated debridements grew *Klebsiella sp*.

**Figure 5:**
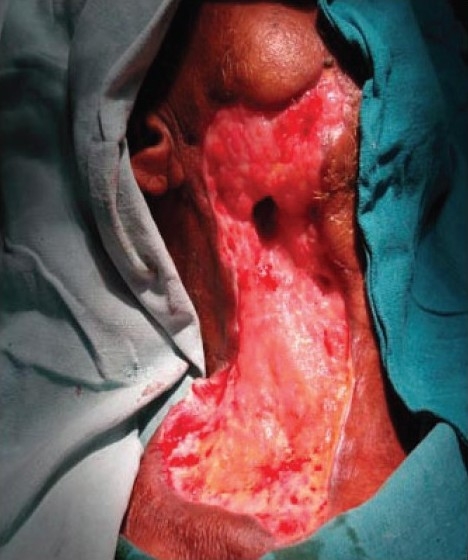
Granulating areas after debridement and dressing

Exploration and decompression of all involved fascial spaces was done. The wound was packed with acriflavine gauze. Intravenous penicillin, gentamicin, and clindamycin were administered initially. Intravenous ciprofloxacin was substituted for penicillin after the culture and sensitivity report became available; gentamycin and clindamycin were continued. Intravenous antibiotics were initially given for 3 weeks during preparation of the graft bed and for a further 2 weeks after skin grafting. Regular dressings with acriflavine-soaked gauze were done till the wound bed was judged to be adequate for skin grafting [Figure 5].

**Figure 6:**
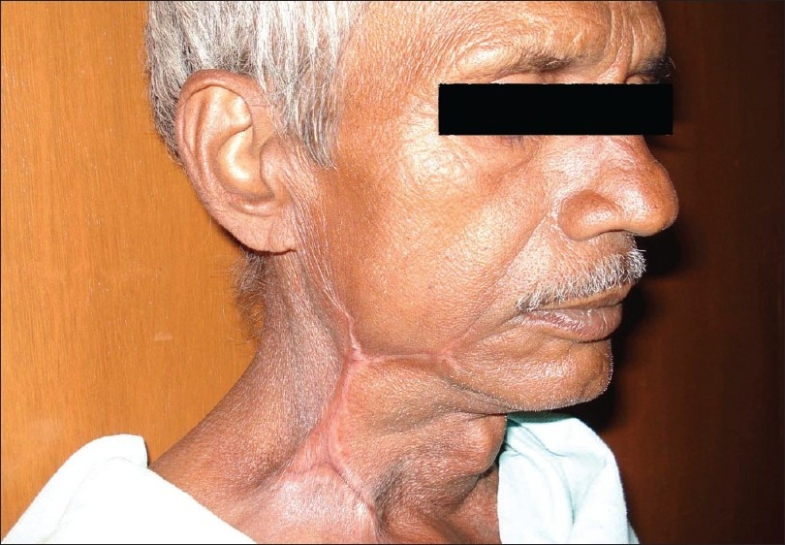
Healed wound after skin grafting

Definitive cosmetic treatment was delayed until the patient's anemia was corrected. He received multiple blood transfusions and amino acid transfusions, and over the next 2 weeks he improved slowly, both hemodynamically and clinically. The raw areas contracted and multiple strips of split-thickness skin grafts were taken from the lateral thigh and grafted on to the areas left exposed by the surgical debridements. He was discharged in a stable condition and is presently doing well [Figure 6].

## DISCUSSION

NF is most common in the perineum, abdominal wall, and extremities. It is less common in the head and neck, especially in the face. The eyelids, scalp, face, and neck are only rarely involved and only a few cases have been reported in the head and neck region.[2,7]

The disease in the head and neck region can occur in two forms, behaving differently in different areas. The two areas are the craniofacial and the cervical.[10] The former involves the scalp and eyelids, where the disease is most commonly caused by trauma (that might be minor)[9,11,12] followed by infection. The organism most commonly isolated in this form of the disease are group A beta-hemolytic streptococci, either alone or in combination with *Staphylococcus aureus.*[12] Only few fatalities have been reported in this group. The latter type classically involves the neck, and the majority of cases follow dental or oropharyngeal infection.

Recently, however, many authors have reported a form of cervicofacial disease which follows dental infection.[11] This type has a rapidly progressive course and, if left untreated, is associated with a mortality of between 22 and 100%.[13] Even minor abrasions, lacerations, burns, and injections can act as a trigger for this serious disease. This was clearly demonstrated in study by Qazi *et al.* which showed that 100% of cases followed an initiating factor.[14] In our case also, though the disease began as a simple dental infection, the triggering factor appears to be the minor trauma resulting from the application of heated medicinal leaves over the face. In NF of the face and scalp, even minor trauma is independently more strongly associated with the condition than infection. In our patient, the color changes in the skin of the face, as also the rapidity of the progression of the disease, increased after the application of the medicinal leaves. Odontogenic deep neck infections and buccal space infections are very common at our rural tertiary care center that caters to a population that is largely from the lower socioeconomic strata; bad oral and general hygiene is common. However, this is the only case of NF in the cervicofacial region that we were able to locate from the records of the last decade. Cases of odontogenic NF are very few as compared to odontogenic abscesses even at centers that deal with large numbers of dental infections.[15] The real trigger for NF is still disputed. Trauma has been implicated as a trigger in the extremities and the face but odontogenic infections head the list in the cervical region. Could it be that trivial trauma inflicted by the patient, or by the surgeon in form of tooth extraction in patients with dental infections, is the real trigger but is usually dismissed as coincidental? NF is very common in the elderly and in patients suffering from chronic debilitating illnesses such as diabetes, chronic renal failure, malignancy, chronic infections, intravenous drug abuse, and immunodeficiency conditions.[14]

Bacteriologic examination in NF usually reveals anaerobes, gram-negative rods, group A beta-hemolytic streptococci, and staphylococcus species. We found *Acinetobacter sp.* in the initial culture from the multiple tissue samples taken. Subsequent culture from separate tissue samples sent after the repeated debridements grew *Klebsiella sp*. There are only few reports of *Acinetobacter* being grown on culture in NF. Our finding was similar to that seen in a series by Qazi *et al.* 2004.[14] The most frequent initial organisms observed in their study were, *Acinetobacter,* streptococcus, *Escherichia coli*, and *S aureus*, with or without anaerobes such as *Bacteroides* or *Peptostreptococci*. Subsequent cultures were more variable and showed predominantly gram-negative organisms and methicillin-resistant *S aureus*. One case showed growth of *Acinetobacter * in the series reported by Panda *et al.*[13] This organism has not been reported in older literature.

*Acinetobacter* is nonfermentative gram-negative coccobacillus that, during the past three decades, has emerged from being an organism of questionable pathogenicity to become an infectious agent of importance in nosocomial infections in hospitals worldwide. *Acinetobacter* infections are more common in tropical countries and the organism is a leading pathogen in wound infections during wars and natural disasters; it has also recently caused multihospital outbreaks in temperate climates. There are at least 21 different *Acinetobacter* genospecies, nine of which have been given formal species names. Within the genus, most of the species of clinical significance belong to the genetically closely related genomic species 2 y(*Acinetobacter baumannii),* 3, and 13 sensu Tjernberg and Ursing (13TU). These genomic species are phenotypically very similar and are collectively known as the *Acinetobacter calcoaceticus-A baumannii* complex (ACB complex) with genomic species 1 (*A calcoaceticus*).[16] The antimicrobial resistance patterns and resistance-harboring genes of *Acinetobacter* species are remarkably distinct according to the genomic species of *Acinetobacter* isolates. In a study, the antimicrobial agents to which *A baumannii* strains were most susceptible were meropenem and imipenem, but the agents to which non-*baumannii Acinetobacter* strains were most susceptible were ciprofloxacin and ampicillin/sulbactam.[16] Based on this evidence, the strain in our case appears to have been non-*baumannii Acinetobacter*. An exhaustive search of PubMed, Medlars, MEDLINE, and Indian Medlars revealed no association between medicinal leaf application and seeding with Acinetobacter *Acinetobacter*. Acinetobacter has been found in traumatic infections during wars, though the source of infection is still uncertain as organism is not very common in the soil. In our case, the pathogen may have been inoculated by the patient by the application of herbal leaves contaminated with *Acinetobacter.*

Imaging studies that may be useful, besides plain soft tissue films of the neck, include chest x-ray to evaluate the mediastinum for widening and to look for pleural effusions and CT scanning, which is probably the single most useful study in the early stages; CT can detect gas in areas inaccessible to palpation, identify areas where infection has spread preoperatively, and can also detect vascular thrombosis, erosion of vessels, or mediastinitis.[17] Our patient presented late with frank areas of necrosis and so immediate surgical intervention was undertaken without CT scanning.

Extensive debridement of all necrotic tissue is the most important part of treatment in these patients. Immediate surgical exploration is indicated in the presence of subcutaneous emphysema, obvious fluctuance, skin necrosis in an area of cellulitis, or rapidly advancing infection despite 24 to 48 h of medical therapy. The areas of fascial necrosis usually extend further than cutaneous involvement.[12]

The complications that have been associated with NF of the head and neck include necrosis of the chest wall fascia, mediastinitis, pleural effusion, pericardial effusion, empyema, airway obstruction, arterial erosion, jugular vein thrombophlebitis, septic shock, lung abscess, carotid artery thrombosis, and DIC.[18]

Several factors have been found to influence survival in NF. In the series reported by Umeda and others, three clinical factors were found to affect mortality: a delay in surgery, the development of mediastinitis, and the presence of medical comorbidities. In another series, the ultimate prognostic factors were old age, female sex (especially with age > 60 years), uncontrolled diabetes mellitus, anemia, coexistent pulmonary diseases, delayed referral (greater than 6 days), and late surgical intervention.[13] Our patient had no comorbidities like diabetes mellitus or renal or pulmonary disease and treatment was initiated within 6–7 days of onset of the facial swelling and, therefore, old age, anemia, and alcoholism, which have been described by some authors as poor prognostic factors, did not have an adverse effect on the outcome.

## CONCLUSION

This report highlights the fact that minor trauma in the form of heat could be a precipitating factor in causing NF in a patient with simple neck space infection of odontogenic origin. Hence the need to create awareness among rural and tribal populations on the danger of inflicting such trauma as a remedy for odontogenic abscesses. A disastrous condition could thus be averted.

This paper also shows the importance of early diagnosis and multiple surgical debridements, which should be undertaken regardless of the extent of cosmetic deformity, in the management of this condition. *Acinetobacter* should be considered as a possible pathogen in patients with NF and care should be taken to select antibiotics that are active against this organism.
